# Advancing terrestrial laser scanning for 3D classification of Spanish moss (*Tillandsia usneoides* L.) using unsupervised, machine learning, and deep learning methods

**DOI:** 10.1038/s41598-026-49230-7

**Published:** 2026-04-20

**Authors:** Alexander J. Gaskins, Jinyi Xia, Inacio T. Bueno, Carlos A. Silva

**Affiliations:** Forest Biometrics, Remote Sensing and Artificial Intelligence Laboratory (Silva Lab), School of Forest, Fisheries, and Geomatics Sciences, University of Florida, Gainesville, FL 32611 USA

**Keywords:** Point cloud, LiDAR, Graph, DBSCAN, Random forest, KPConv, PointNet++, Computational biology and bioinformatics, Ecology, Ecology, Plant sciences

## Abstract

Quantifying the distribution of Spanish moss (*Tillandsia usneoides* L.) is challenging because it grows suspended from high tree branches, limiting manual sampling. Terrestrial laser scanning (TLS) provides a non-destructive means of capturing vegetation structure in three dimensions. However, no established methods exist for identifying Spanish moss from TLS data. We evaluated five classification methods for distinguishing Spanish moss in TLS-derived point cloud data: Graph, DBSCAN, Random Forest (RF), Kernel Point Convolution (KPConv), and PointNet++. PointNet++ achieved the highest accuracy (81%), followed by DBSCAN (70%), KPConv (61%), RF (54%), and Graph (52%). Unsupervised methods required minimal computational resources (2–3 min, 8–16 GB memory) without training. RF required 3 h for training, 8 for prediction with 1024 GB memory. Deep learning methods required substantially more: KPConv needed 60 h for training, 4 for prediction (256 GB), while PointNet++ required 48 h for training, 1 for prediction (128 GB). Agreement was lowest in the central and upper canopy due to occlusion. Surface variation, PCA1, and verticality contributed most to accurate predictions. These results demonstrate the feasibility of using TLS and advanced classification methods for non-destructive Spanish moss mapping and highlight the accurate classification ability of PointNet++ for future biomass estimation at landscape scales.

## Introduction

Spanish moss (*Tillandsia usneoides* L.) is a widespread epiphytic bromeliad native to the Americas, ranging from the southeastern United States to Argentina. It grows in long, gray, draping strands that hang from the tree branches, absorbing nutrients and water directly from the atmosphere^1,2^. Spanish moss provides important habitat functions as it is used by birds for nest building^3^ and hosts a diverse assemblage of insects and spiders^4,5^. It also plays unique ecological roles in forest canopies once it influences water interception^6^, tolerates extreme stress through Crassulacean Acid Metabolism^7^, and contributes to carbon storage, with dry biomass containing approximately 43% carbon^8,9^. Furthermore, its sensitivity to airborne compounds makes it an effective biomonitor for nitrogen and carbon emissions^10^ as well as urban pollution^11–13^. These ecological functions of Spanish moss underscore the need to accurately quantify the biomass of Spanish moss.

Spanish moss typically grows high in the canopy^14^, making manual collection of samples for biomass estimation difficult, time-consuming, and often invasive^15^. This destructive sampling also introduces additional biases, as it can alter the nutrient and moisture content of the specimens, remove the ability to measure samples again, disturb associated organisms (insects and birds), and fail to capture spatial patterns at the individual or ecosystem level^16^. With increasing emphasis on quantifying all carbon pools in forest systems, not just trees but also epiphytic and non-vascular plants, there is an urgent need for a reliable, non-destructive method to estimate the biomass of Spanish moss^17^. Such methods are critical for integrating this species into forest carbon accounting frameworks^18^ and for improving understanding of its contributions to carbon sequestration and biodiversity-related ecosystem functions.

Remote sensing technologies such as terrestrial laser scanning (TLS) provide a promising solution for quantifying forest attributes, achieving an RMSE of 1–2 cm^19^, and can hold particular potential for epiphytic vegetation beyond trees^20^. TLS captures high-density, three-dimensional point cloud data of forest structures and has been widely applied to estimate tree height with an RMSE of 0.95 m^21^, diameter at breast height at an R^2^ of 0.98 and RMSE of 1.59 cm^22^, crown architecture with accuracies above 80% and R^2^ values greater than 0.64^23^, and aboveground biomass with an RMSE of 0.78^24^. As a non-destructive, repeatable, and comprehensive sampling method, TLS enables detailed structural characterization of forest canopies with R^2^ values greater than 0.7^25^.

Recent studies have further extended TLS applications to leaf–wood separation using unsupervised (data classified based on similarity without prior labels) and supervised methods (data classified based on labeled training data to learn class boundaries)^26,27^. Examples of unsupervised methods include density-based spatial clustering of applications with noise (DBSCAN) and graph methods. They have the advantage of automatically identifying natural clusters in point clouds and requiring little prior information, which is often scarce in forestry applications^28^. However, they can be sensitive to parameter settings, frequently struggle with noise, and may fail to capture fine structural details in complex canopy environments^29^. Other studies have also shown that their generalization ability across different tree species remains limited and requires improvement^30^. Even when high accuracies are reported, errors are common in dense canopies where leaves, twigs, and branches overlap, leading to occlusion and preventing TLS from fully capturing interior structures^31^.

On the other hand, supervised methods have shown strong performance in TLS classification tasks^32^. Machine learning methods such as Random Forest (RF) generally achieve better performance than unsupervised methods and are valued for their robustness across sites and datasets^33,34^. Deep learning architectures, including Kernel Point Convolution (KPConv) and PointNet++, are among the most effective architectures for 3D point cloud analysis, both of which have demonstrated competitive performance across standard benchmarks^35,36^. These methods are particularly effective in complex canopy environments, as they learn directly from raw point clouds without the need for handcrafted features^37^. For example, KPConv distributes kernel points on a sphere, enabling the learning of anisotropic features that can be discriminative for separating leaf and wood structures^38^. However, the advantages of supervised methods come with trade-offs: they require large, manually labeled datasets, and computational demands increase substantially from machine learning to deep learning^39^. Moreover, despite improvements in accuracy, residual misclassification in dense, overlapping canopy regions remains an unresolved challenge^40,41^.

Despite these advances, TLS classification accuracy remains constrained by canopy complexity and has never been investigated for epiphytic species to date. No study has applied TLS-based classification to Spanish moss, despite its prevalence in forest canopies and ecological importance. Moreover, no systematic comparison of unsupervised, machine learning, and deep learning methods has been conducted for epiphyte classification, leaving it unclear which methods are most effective for these canopy components. This study addresses this gap by evaluating five classification methods for detecting and segmenting Spanish moss from TLS data collected on an urban environment at the University of Florida campus: two unsupervised (DBSCAN and Graph) and three supervised (RF, PointNet++, and KPConv). Specifically, we aim to (1) assess the feasibility of using TLS for non-destructive mapping of Spanish moss, (2) compare classification accuracy and processing efficiency across methods, and (3) identify the most effective method for future biomass estimation. By establishing the first benchmark for Spanish moss classification, our study provides a foundation for quantifying its carbon storage potential and better incorporating epiphytes into forest carbon accounting.

## Materials and methods

### Study area

**Fig. 1:**
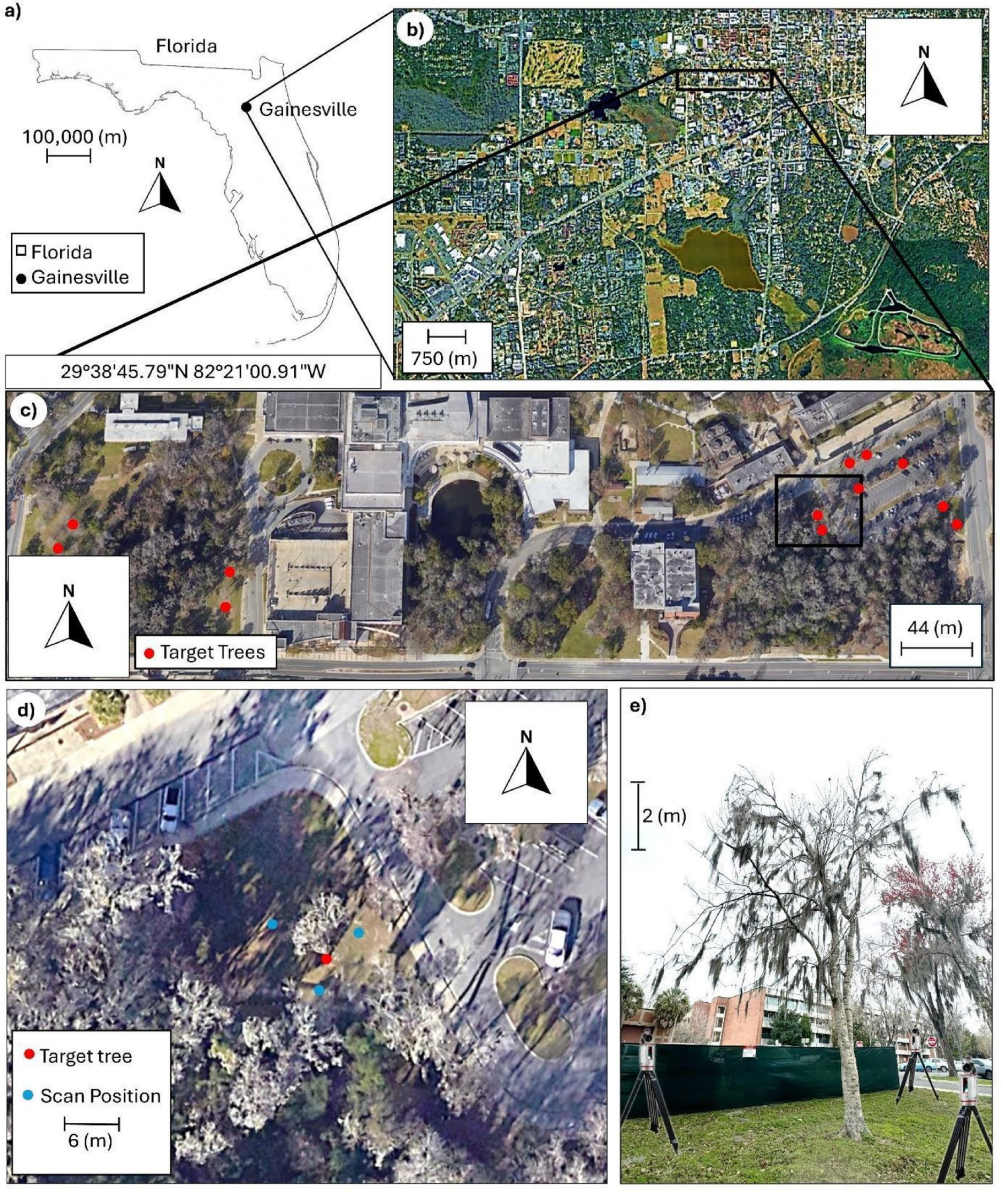
Study area and data collection. (**a**) Location of the study area in the state of Florida; (**b**) location in the city of Gainesville; (**c**) locations of target trees on the University of Florida campus; (**d**) schematic of three scan positions per tree; and (**e**) example of a target tree used for TLS data collection. Imagery/maps for (**b**), (**c**), and (**d**) from Google Imagery available in QGIS 3.44.5.

Data collection was conducted on the University of Florida’s campus in Gainesville, Florida (Fig. 1a,b), which has a high presence of Spanish moss. The area is primarily urban but includes wooded areas with diverse tree species that provide suitable habitats for Spanish moss. It is regularly maintained through arboricultural practices, including branch removal, which can reduce the local abundance of Spanish moss. The study area lies at an elevation of approximately 50 m^42^ and experiences a humid subtropical climate with a mean annual precipitation of 1330 mm and an average annual temperature of 24 °C^43^.

### TLS data collection and processing

TLS data were collected in early spring 2024 using a Riegl VZ-400i terrestrial laser scanner coupled with a NIKON D850 45.7-megapixel digital camera and a differential GNSS RTK receiver. RGB information was used exclusively for visualization purposes and was not incorporated into the classification workflow to ensure methodological reproducibility across TLS systems, as not all instruments provide RGB data. Twelve trees of multiple species, including chinaberry *(Melia azedarach*), red maple (*Acer rubrum*), white ash (*Fraxinus americana*), sweetgum (*Liquidambar styraciflua*), and crepe myrtle (*Lagerstroemia indica*), were selected based on a visible gradient of Spanish moss coverage, from low to high density (Fig. 1c). Data collection occurred prior to leaf emergence in deciduous trees, ensuring minimal foliage interference. For each tree, three scans were acquired from different positions to reduce occlusion (Fig. 1d,e).

**Fig. 2:**
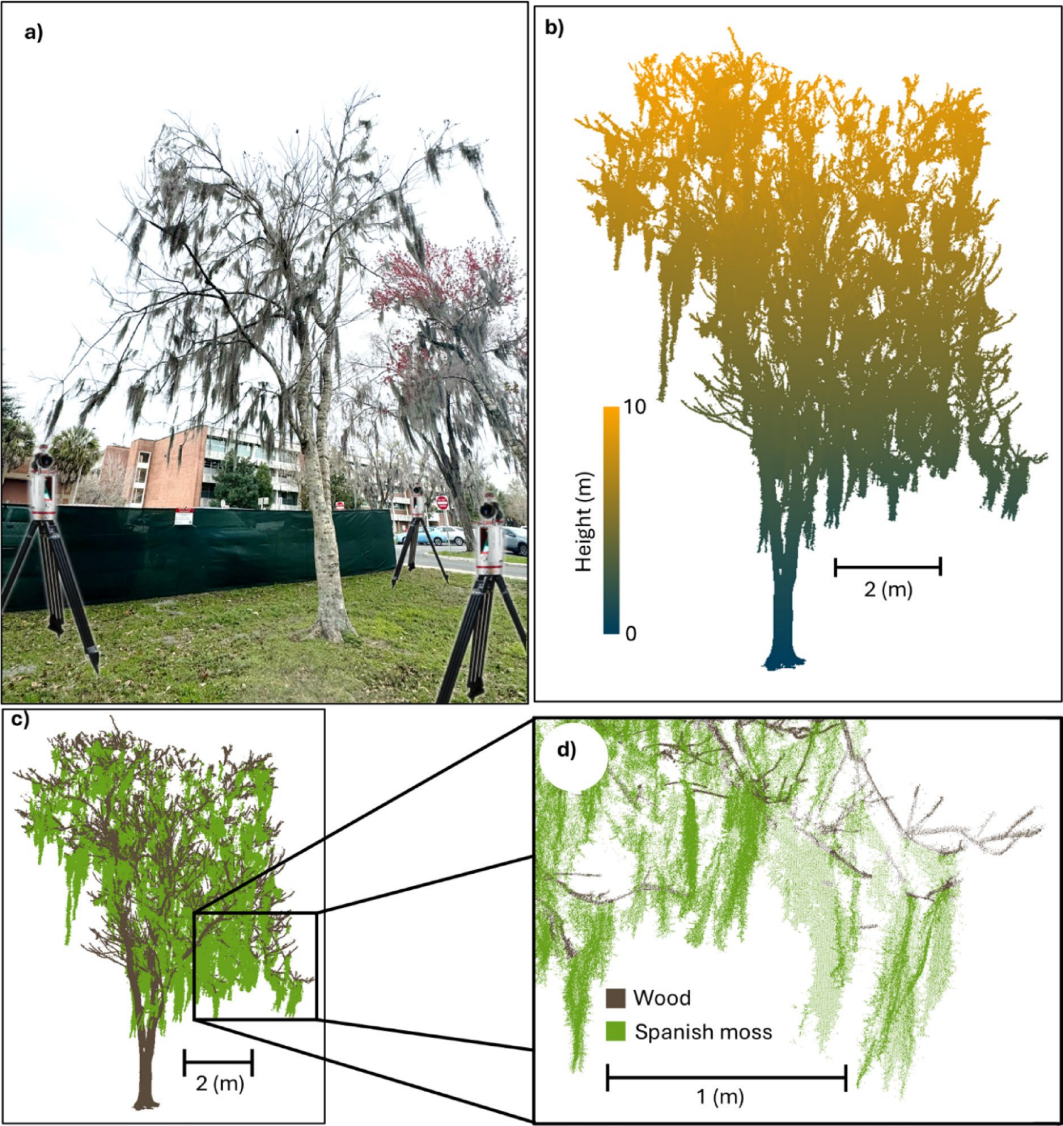
3D point cloud data collection. (**a**) Riegl VZ-400i scanner; (**b**) Preprocessed 3D point cloud; (**c**) Manually classified 3D point cloud; (**d**) Zoom in on a manually classified 3D point cloud.

Point cloud preprocessing, including noise removal, registration, and clipping of individual trees, was performed using RiSCAN Pro (version 2.14.1)^44^ and CloudCompare v2.12^45^. Figure 2a shows the data collection setup, and Fig. 2b shows an example of the resulting 3D point cloud data.

For creating the reference datasets, Spanish moss was manually separated from non-Spanish moss structures in CloudCompare. Two classes were defined: Spanish moss, consisting of the irregular, filamentous clumps of Spanish moss hanging from branches, and non-Spanish moss, which included the trunk, branches, and all remaining structures. Approximately 8 h per tree were required for manual classification during reference data collection. Across all trees, the proportion of Spanish moss ranged from 3.8 to 81.2%, with total point counts per cloud varying between 1.5 million and 10.1 million. To address class imbalance, additional moss samples were generated by duplicating point clouds with sparse Spanish moss coverage and applying geometric transformations, such as rotation and scaling.

### Point-wise geometric feature extraction

**Fig. 3:**
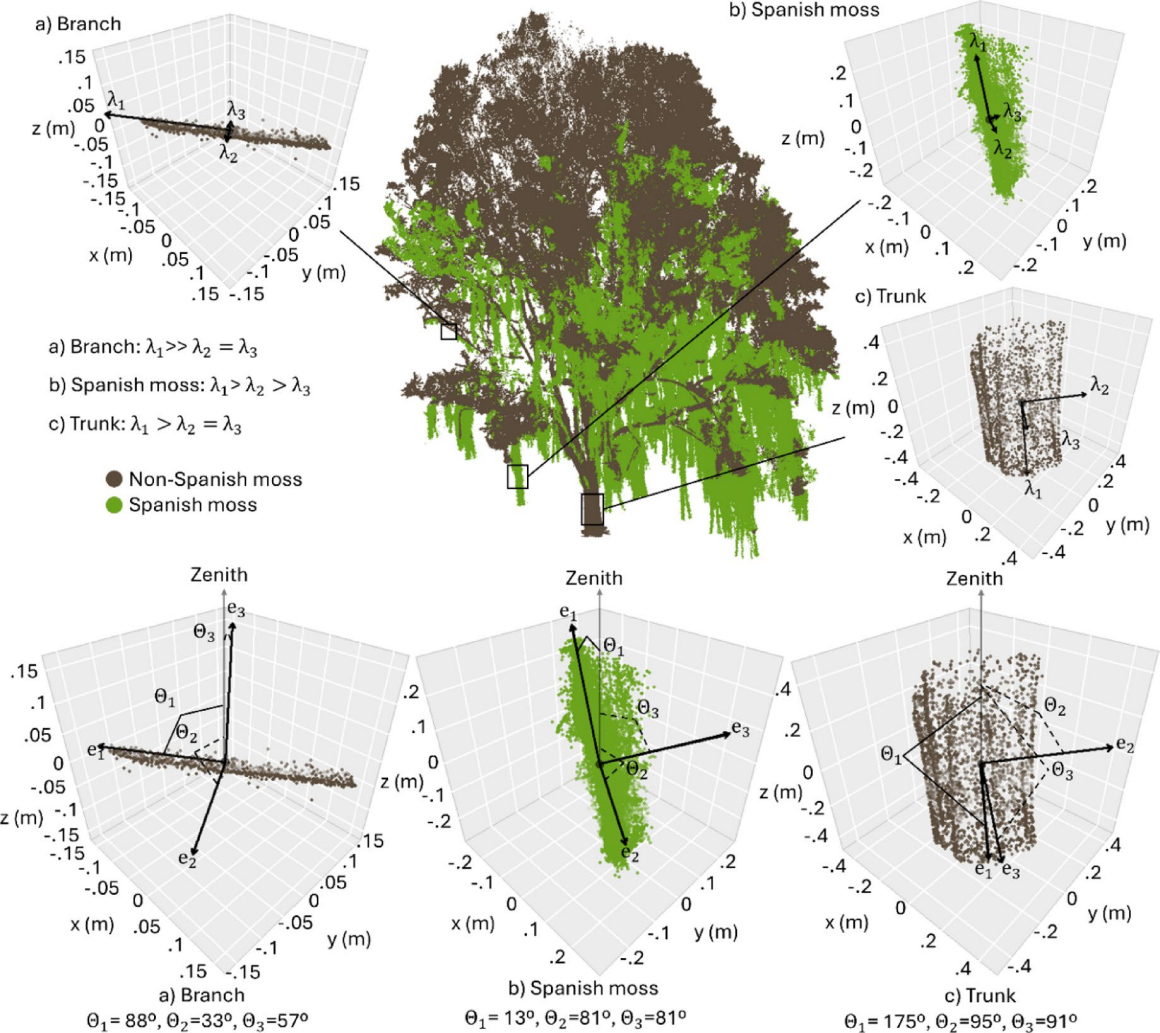
Illustration of eigenvalues (λ) and eigenvectors (Θ) derived from PCA of local point neighborhoods: (**a**) eigenvalues (λ₁ > λ₂ ≈ λ₃) and (**d**) eigenvectors corresponding to branch-like structures; (**b**) eigenvalues (λ₁ ≈ λ₂ ≈ λ₃) and (**e**) eigenvectors corresponding to Spanish moss structures; and (**c**) eigenvalues (λ₁ ≈ λ₂ > λ₃) and (**f**) eigenvectors corresponding to trunk-like structures.

Fourteen geometric features were computed as inputs for the classification methods (Table 1). For each point, a local neighborhood was defined, and a covariance matrix of the centered 3D coordinates was constructed. Principal Component Analysis (PCA) was then applied to extract eigenvalues (λ) and eigenvectors (Θ), which characterize the magnitude and orientation of geometric variation within the neighborhood (Fig. 3). The features, defined from combinations of λ and Θ, were assigned to each point.

**Table 1:** Geometric features and their respective equations are used as input for classification methods. λ: PCA eigenvalues; λ_i_: normalized eigenvalue; ${V}_{z}$: z-component of the principal eigenvector.

Geometric feature	Equation	Geometric feature	Equation
Eigenvalue sum	${\lambda }_{1}+{\lambda }_{2}+{\lambda }_{3}$	PCA2	${\lambda }_{2} / ({\lambda }_{1}+{\lambda }_{2}+{\lambda }_{3})$
Omnivariance	${(\lambda }_{1}\times {\lambda }_{2}{{\times \lambda }_{3})}^{1/3}$	Surface variation	${\lambda }_{3} / ({\lambda }_{1}+{\lambda }_{2}+{\lambda }_{3})$
Eigenentropy	$-\sum_{i=1}^{3}{\lambda }_{i}ln({\lambda }_{i})$	Sphericity	${\lambda }_{3} / {\lambda }_{1}$
Anisotropy	${(\lambda }_{1}-{\lambda }_{3})/{\lambda }_{1}$	Verticality	$1-|{V}_{z}|$
Planarity	${(\lambda }_{2}-{\lambda }_{3})/{\lambda }_{1}$	Nx	x normal vector
Linearity	${(\lambda }_{1}-{\lambda }_{2})/{\lambda }_{1}$	Ny	y normal vector
PCA1	${\lambda }_{1} / ({\lambda }_{1}+{\lambda }_{2}+{\lambda }_{3})$	Nz	z normal vector

### Spanish moss classification methods

**Fig. 4:**
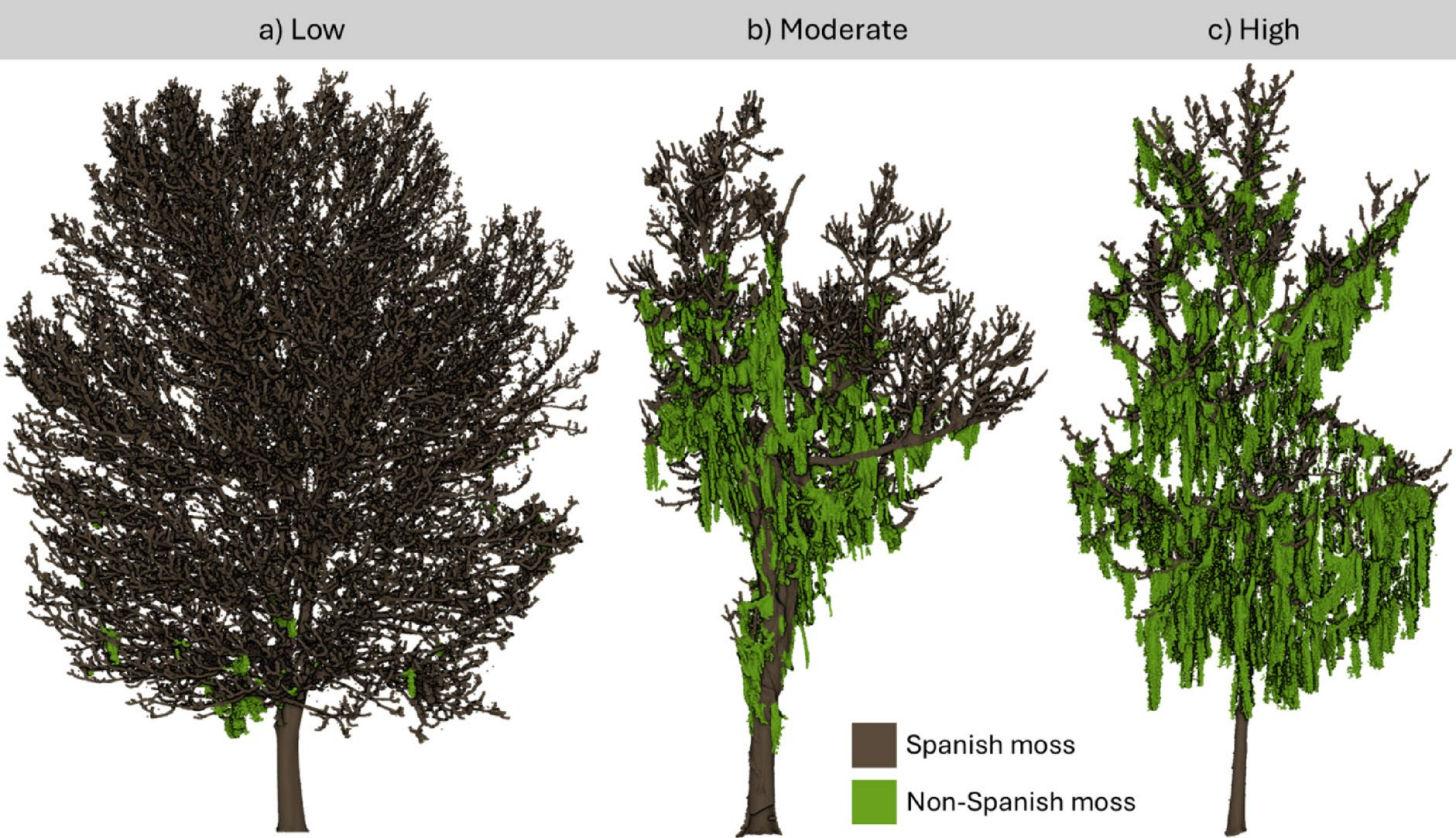
Examples of Spanish moss density classes: (**a**) low, (**b**) moderate, and (**c**) high.

The dataset comprised 50,400,313 points, sourced from 12 individual tree point clouds containing Spanish moss. The data were divided into training, validation, and test datasets following standard machine learning practice, with a target allocation of 70%, 15%, and 15%, respectively. Stratification was performed based on Spanish moss density to balance low, moderate, and high classes (Fig. 4); however, because trees contributed varying numbers of points, exact targets were not achievable, resulting in final proportions of 66.5% (33,499,055 points) for training, 15.2% (7,676,827 points) for validation, and 18.3% (9,224,431 points) for testing. Five methods were applied, grouped into unsupervised (Graph and DBSCAN) and supervised methods (RF, KPConv, and PointNet + +) as follows:

#### Graph

**Fig. 5:**
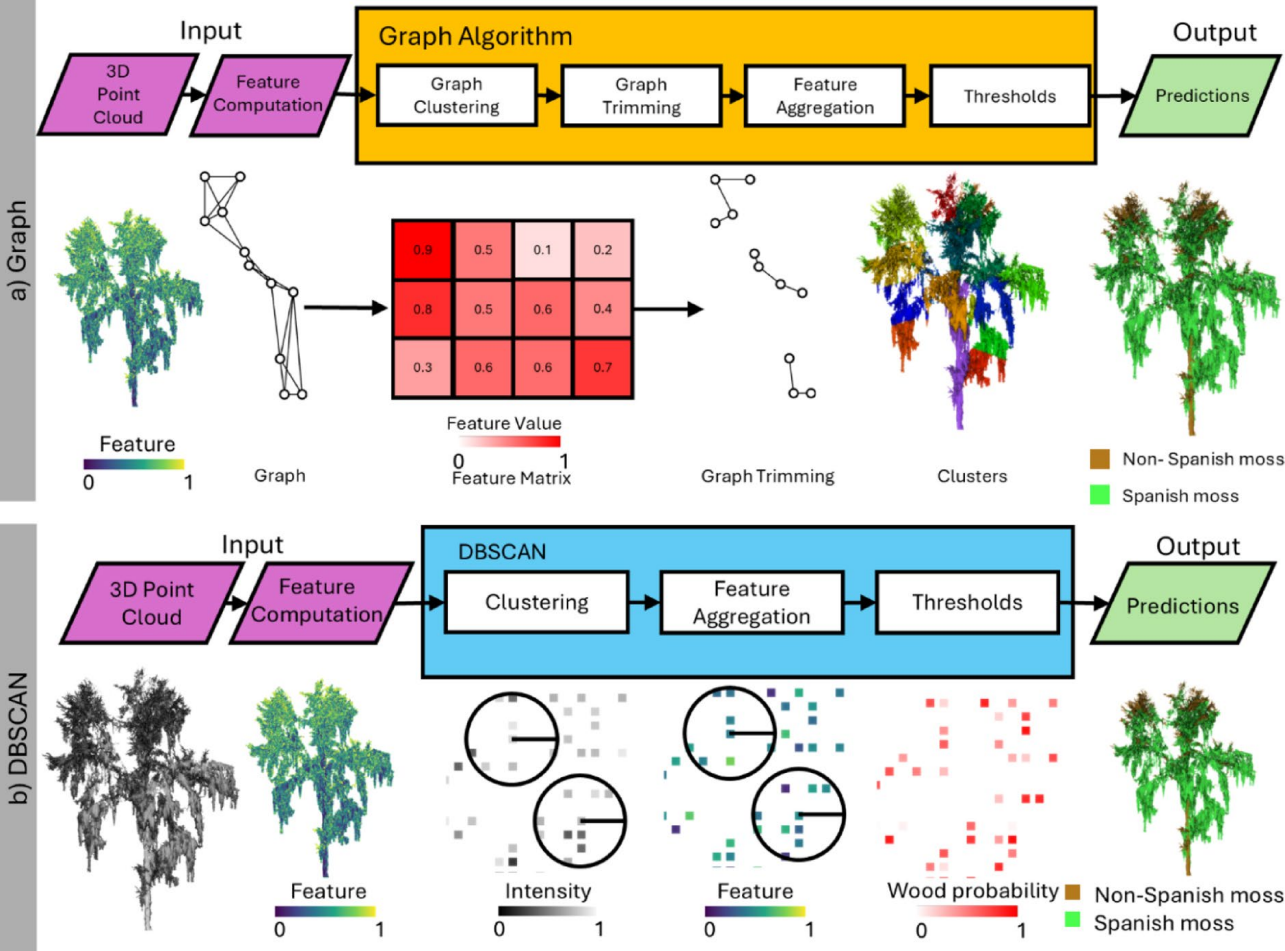
The workflows for the unsupervised methods. (**a**) Graph architecture workflow; (**b**) DBSCAN architecture workflow.

Graph used a k-nearest neighbor graph made by connecting each point to its 10 nearest neighbors, with a 5 cm distance threshold set in this study. Edges between points whose Surface variation values exceeded a similarity threshold of 0.05 were trimmed. Remaining connected points were clustered, and those smaller than or equal to the minimum cluster size of 1 were relabeled according to their nearest neighbor. Average feature values and probability of a point being non-Spanish moss^46^ were calculated for each cluster. To further refine classification, points below 50 cm on the z-axis threshold were assigned as non-Spanish moss points. Finally, a threshold of 0.1 was used, with points below this probability of a point being non-Spanish moss threshold classified as non-Spanish moss (Eq. 1)^46^. A visual overview of the Graph architecture is seen in Fig. 5a. Graph was implemented using the R package lidUrb^47^.

#### DBSCAN

DBSCAN^28^ started by assigning an initial label of Spanish moss to points that had a verticality of greater than 0.8. Clustering was applied using a neighborhood radius of 1 cm. Geometric feature values were aggregated for each cluster to characterize overall geometry, and the probability of non-Spanish moss was computed. Clusters smaller than the minimum size of 5 points inherited the same probability as their nearest valid neighbor. In addition, any cluster that contained points below a 50 cm z-axis threshold was reclassified as non-Spanish moss. Final labels (Spanish moss or non-Spanish moss) were assigned using a probability threshold of 0.001 for non-Spanish moss (Eq. 1)^46^. A visual workflow of the DBSCAN method is shown in Fig. 5b. DBSCAN was implemented from the R package lidUrb^47^.

1 $$S=\left\{{pr\in \left[0,1\right]}^{C}|\sum_{k\in C}{pr}_{k}=1\right\}$$

#### Random forest

**Fig. 6:**
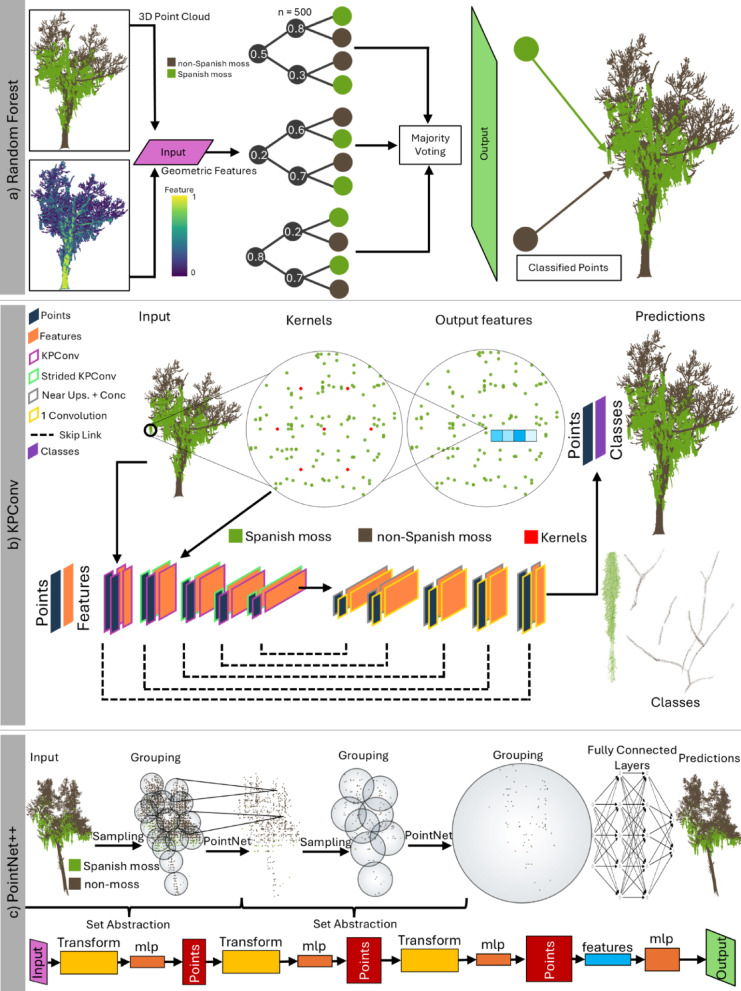
Workflows for supervised methods: (**a**) RF architecture workflow; (**b**) KPConv architecture workflow; (**c**) PointNet ++ architecture workflow.

In RF, point clouds were subsampled with a voxel size of 50 cm × 50 cm × 50 cm and a target density of two points per voxel. 500 decision trees were made by feature thresholds that were sampled randomly without replacement based on the points’ feature values to create an output based on the most popular classification decision made^48^. Multiple decision trees voted on unseen test data to determine how to classify each point. A visual workflow of the RF process is seen in Fig. 6a. randomForest is from the R package by Liaw and Wiener^49^, based on Breiman’s RF model^50^.

#### KPConv

KPConv required preprocessing that the other methods did not, as it firstly requires normals to be computed, which was done using CloudCompare®, and the data to be arranged as x, y, z, normals x, normals y, normals z, and classification. To start, KPConv used 6 workers and a batch size of 32 to apply grid subsampling on the points to reduce their number with a first subsampling of 0.005. Next, it constructed neighborhoods by grouping nearby points, forming a tensor that was used to build the model architecture (Fig. 6b). An encoder-decoder framework extracted hierarchical features that were used to make predictions that were compared to the true labels, which calculated the loss function, and the model adjusted the positions of kernels to maximize the method’s ability to learn. Adaptive Moment Estimation (Adam), with a batch normalization policy utilizing a batch normalization momentum of 0.1, batch normalization decay of 0.9999, and batch normalization clip of 0.01, optimized the learning rate by utilizing step decay with a 20 decay step and backpropagation to alter kernel weights to improve performance. KPConv then validates against the validation dataset, where the learning scheduler may adjust the learning rate based on the validation performance. A visual representation of the KPConv workflow is viewable in Fig. 6b. KPConv is from the Python package of the same name^35^.

#### PointNET++

PointNet++^36^ started with subsampling points into regions with a minimum spacing of 3 cm, 750 minimum points per region, and 20,000 maximum points per region. Training started with a batch size of 14 by using farthest point sampling that picks evenly distributed centroid points. From these centroids, local neighborhoods of nearby points were created by the process of ball query, which determined neighborhoods. In each neighborhood, a shared multilayer perceptron was applied independently to each point within the neighborhood, performing a series of linear transformations followed by nonlinear activations to extract features from each point’s coordinates. Within each local neighborhood, max pooling aggregated the pointwise features into a fixed-size vector, summarizing the most prominent local characteristics. PointNet++ then used set abstraction that used farthest point sampling, multilayer perceptrons, and max pooling to make hierarchical features at multiple scales, producing a local to global feature hierarchy. Local features were hierarchically combined through multiple set abstraction layers to form global features. Feature propagation layers upsampled features by interpolating coarse-level features and combining them with skip-connected features from earlier layers. Then predictions were made on the training dataset in the training phase and the validation phase, where the batch size was 14. Loss was computed by comparing the manually classified labels and the predictions from the model. Weights were adjusted by backpropagation using gradient descent at a learning rate of 0.00005. A visual representation of the PointNet++ workflow is viewable in Fig. 6c. The parameters used for each method are summarized in Table 2.

**Table 2:** Parameter table for all methods.

Method	Parameter	Value
Graph	Number of neighbors	10 points
	Neighborhood radius	5 cm
	Geometric feature similarity threshold	0.05
	Minimum cluster size	1 point
	z axis reclass threshold	50 cm
	Prediction threshold	0.1
DBSCAN	Initial label geometric feature	verticality
	Initial label geometric feature threshold	0.8
	Neighborhood radius	1 cm
	Minimum cluster size	5 points
	z axis reclass threshold	50 cm
RF	Subsample voxel size	50 cm
	Subsample target density	2 points/voxel
	Number of decision trees	500
KPConv	Workers	6
	Batch size	32
	First subsampling	0.005
	Optimization algorithm	Adam
	Batch normalization momentum	0.1
	Batch normalization decay	0.9999
	Batch normalization clip	0.01
	Decay step	20
PointNet++	Minimum spacing	3 cm
	Minimum points per region	750
	Maximum points per region	20,000
	Training batch size	14
	Validation batch size	14
	Learning rate	0.00005

### Classification assessment

**Fig. 7:**
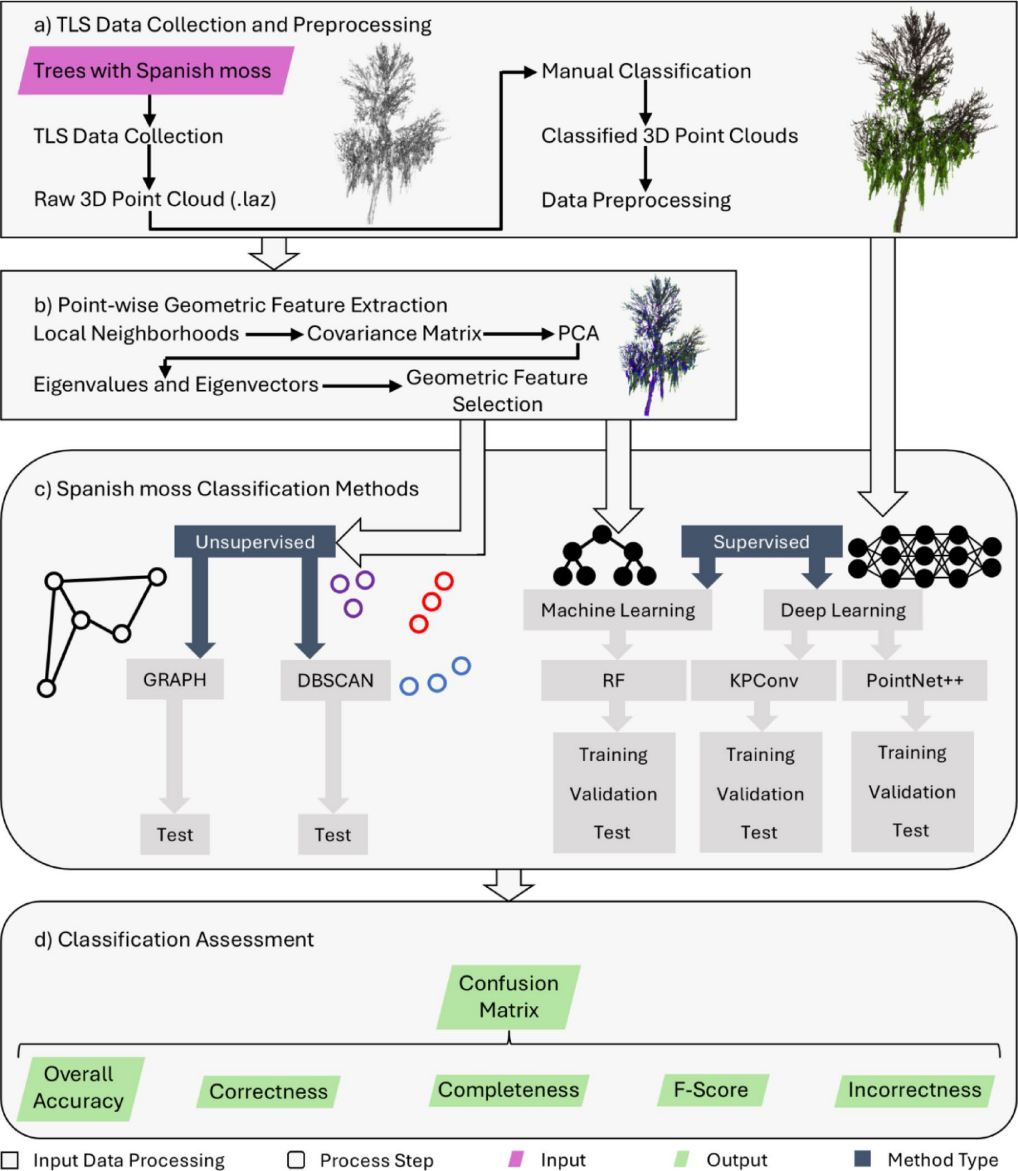
Workflow from data acquisition to evaluation. (**a**) TLS data collection and preprocessing, including point cloud acquisition, manual classification, and preprocessing; (**b**) point-wise geometric feature extraction, from neighborhood establishment to geometric feature selection; (**c**) Spanish moss classification methods, including unsupervised and supervised methods; (**d**) classification assessment, including confusion matrix calculation and derived performance metrics.

Model performance was evaluated using confusion matrices generated with the caret package^51^, comparing manually classified labels with model predictions. The confusion matrices were defined in terms of true positives (TP, Spanish moss correctly classified as Spanish moss), true negatives (TN, non-Spanish moss correctly classified as non-Spanish moss), false positives (FP, non-Spanish moss incorrectly classified as Spanish moss), and false negatives (FN, Spanish moss incorrectly classified as non-Spanish moss). From these, several evaluation metrics were derived: overall accuracy (the proportion of correctly classified points), correctness or precision (the proportion of predicted Spanish moss that are actually Spanish moss), completeness or recall (the proportion of actual Spanish moss correctly identified, indicating sensitivity to under-segmentation), F-score (the harmonic mean of correctness and completeness), and incorrectness (the proportion of Spanish moss points misclassified as non-Spanish moss)(Eqs. (2–6)). The overall workflow from data acquisition to evaluation is illustrated in Fig. 7.

2 $$Overall ~accuracy=\frac{{T}_{P}+{T}_{P}}{{T}_{P}+{T}_{N}+{F}_{P}+{F}_{N}}$$

3 $$Correctness=\frac{{T}_{P}}{{T}_{P}+{F}_{P}}$$

4 $$Completness=\frac{{T}_{P}}{{T}_{P}+{F}_{N}}$$

5 $$F ~score=2\times \frac{Completeness\times Correctness}{Completeness+Correctness}$$

6 $$Incorrectness=1-\frac{{T}_{N}}{{T}_{N}+{F}_{N}}$$

## Results

### Overall method performance and assessment

**Fig. 8:**
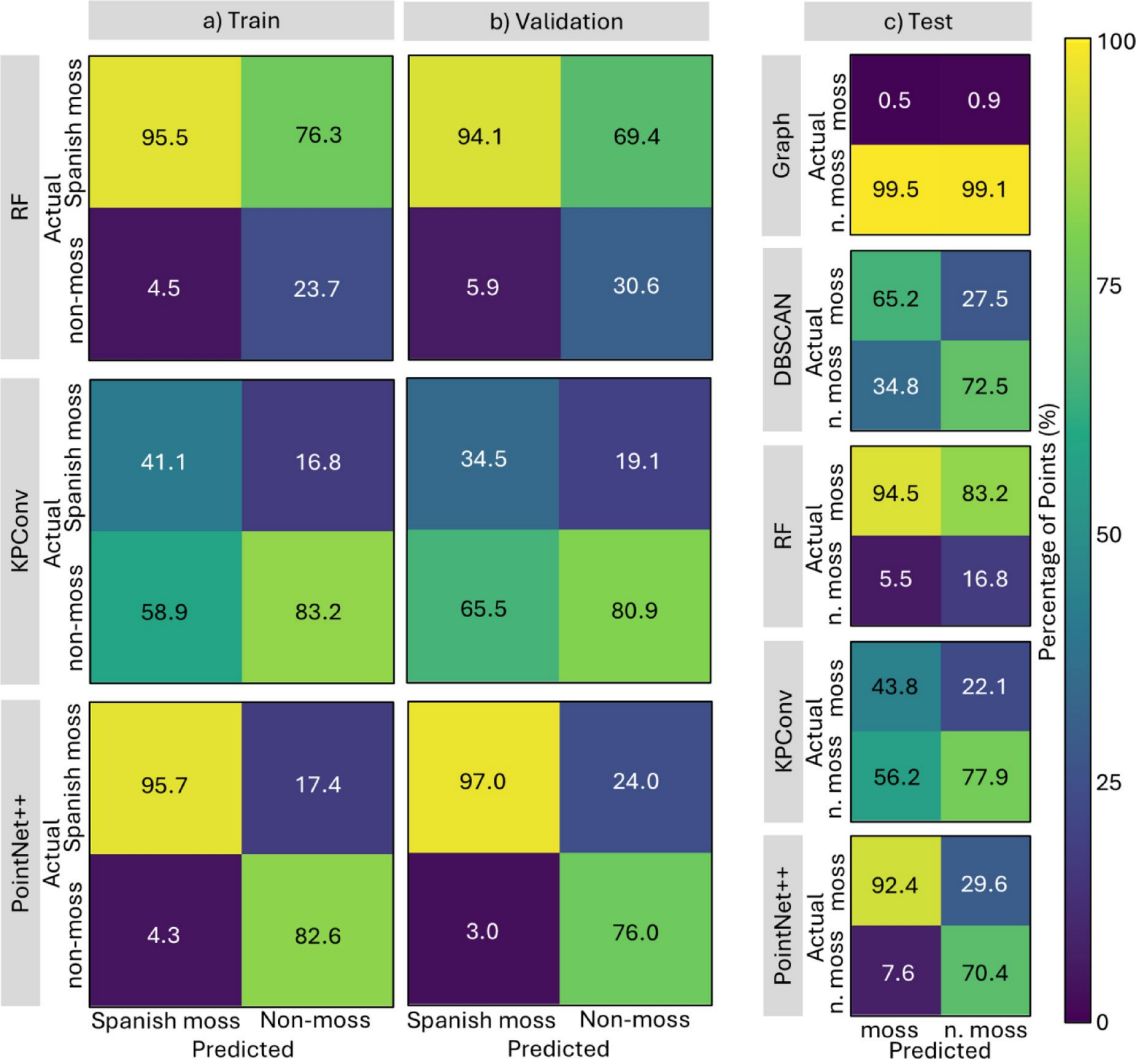
Confusion matrices for supervised and unsupervised methods: (**a**) Training, (**b**) Validation, and (**c**) Test datasets.

Quantitative metrics confirm the visual patterns (Fig. 8; Tables 3, 4). Correct classification of Spanish moss ranged from 10.9% (Graph) to 97% (PointNet + +). Training datasets (Fig. 8a) yielded higher accuracy than validation (Fig. 8b) and test datasets (Fig. 8c). KPConv consistently overestimated moss, with 56.2–65.5% of non-Spanish moss points classified as moss. On the test dataset, Graph showed a similar problem (89.1% misclassified) and DBSCAN to a lesser extent (34.8% misclassified).

**Table 3:** Training and validation evaluation metrics for supervised methods.

Dataset	Metrics	Machine learning	Deep learning	Dataset
		RF	KPConv	PointNet++
Training	Overall accuracy	0.57	0.65	0.91
	Completeness	0.95	0.41	0.90
	Correctness	0.49	0.60	0.84
	F-Score	0.59	0.44	0.87
	Incorrectness	0.76	0.39	0.05
Validation	Overall accuracy	0.66	0.55	0.88
	Completeness	0.94	0.32	0.97
	Correctness	0.61	0.62	0.82
	F-Score	0.73	0.42	0.89
	Incorrectness	0.70	0.50	0.07

**Table 4:** Test dataset evaluation metrics for all methods.

Metrics	Unsupervised		Supervised		
			Machine learning	Deep learning	
	Graph	DBSCAN	RF	KPConv	PointNet++
Overall accuracy	0.52	0.70	0.54	0.61	0.81
Completeness	0.00	0.65	0.94	0.44	0.92
Correctness	0.32	0.69	0.51	0.65	0.74
F-Score	0.01	0.71	0.66	0.52	0.82
Incorrectness	0.48	0.31	0.83	0.40	0.09

In training (Table 3), PointNet++ achieved the highest overall accuracy (91%) and F-score (0.87), while RF and KPConv showed moderate performance. Validation metrics revealed a similar ranking, though RF outperformed KPConv. In the test dataset (Table 4), PointNet++ again performed best (81% accuracy), followed by DBSCAN (70%), KPConv (61%), RF (54%), and Graph (52%).

**Fig. 9:**
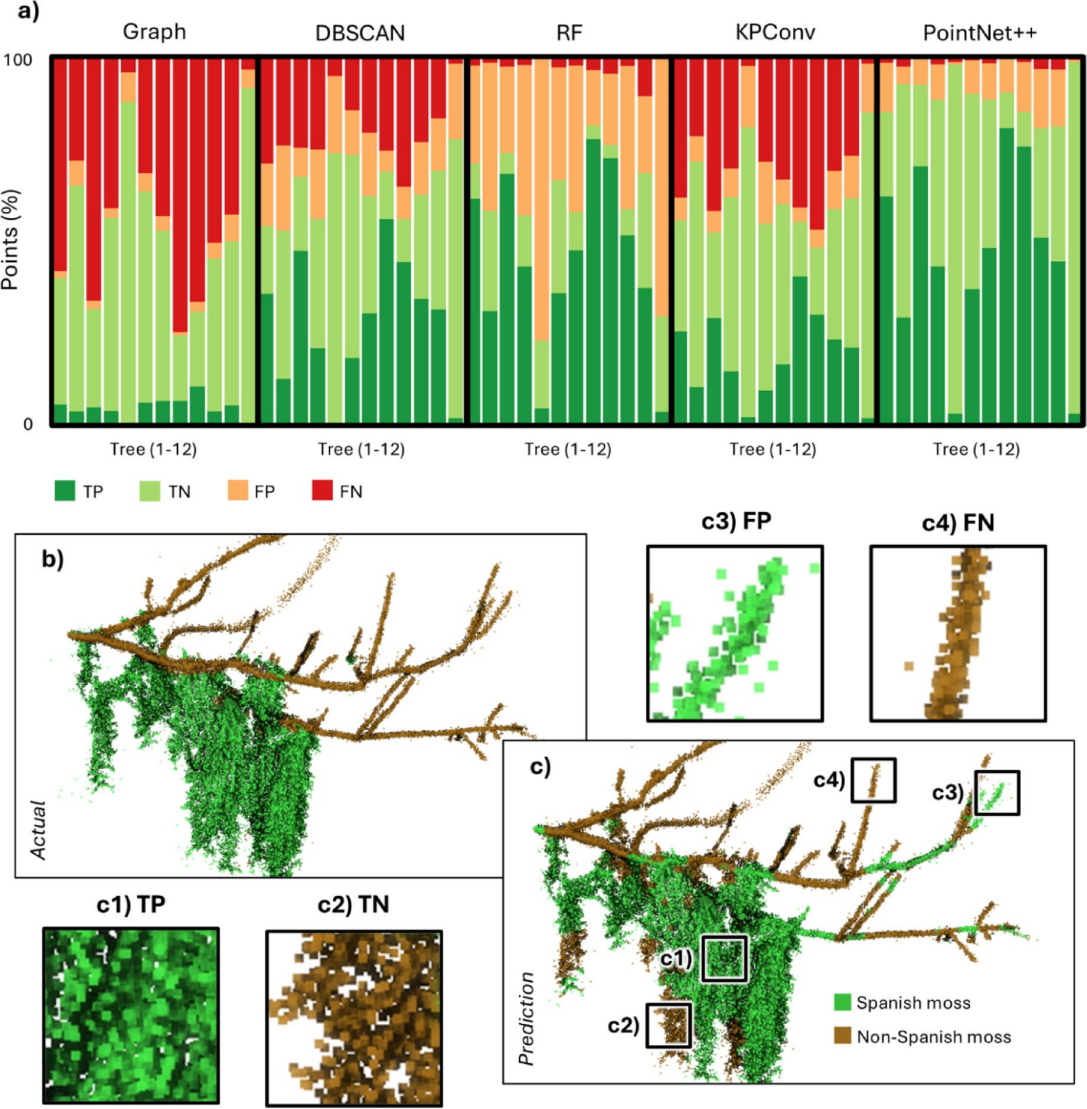
Confusion composition across methods: (**a**) TP/TN/FP/FN proportions by tree; (**b**) Reference labels; (c1–c4) Representative TP, TN, FP, and FN examples.

**Fig. 10:**
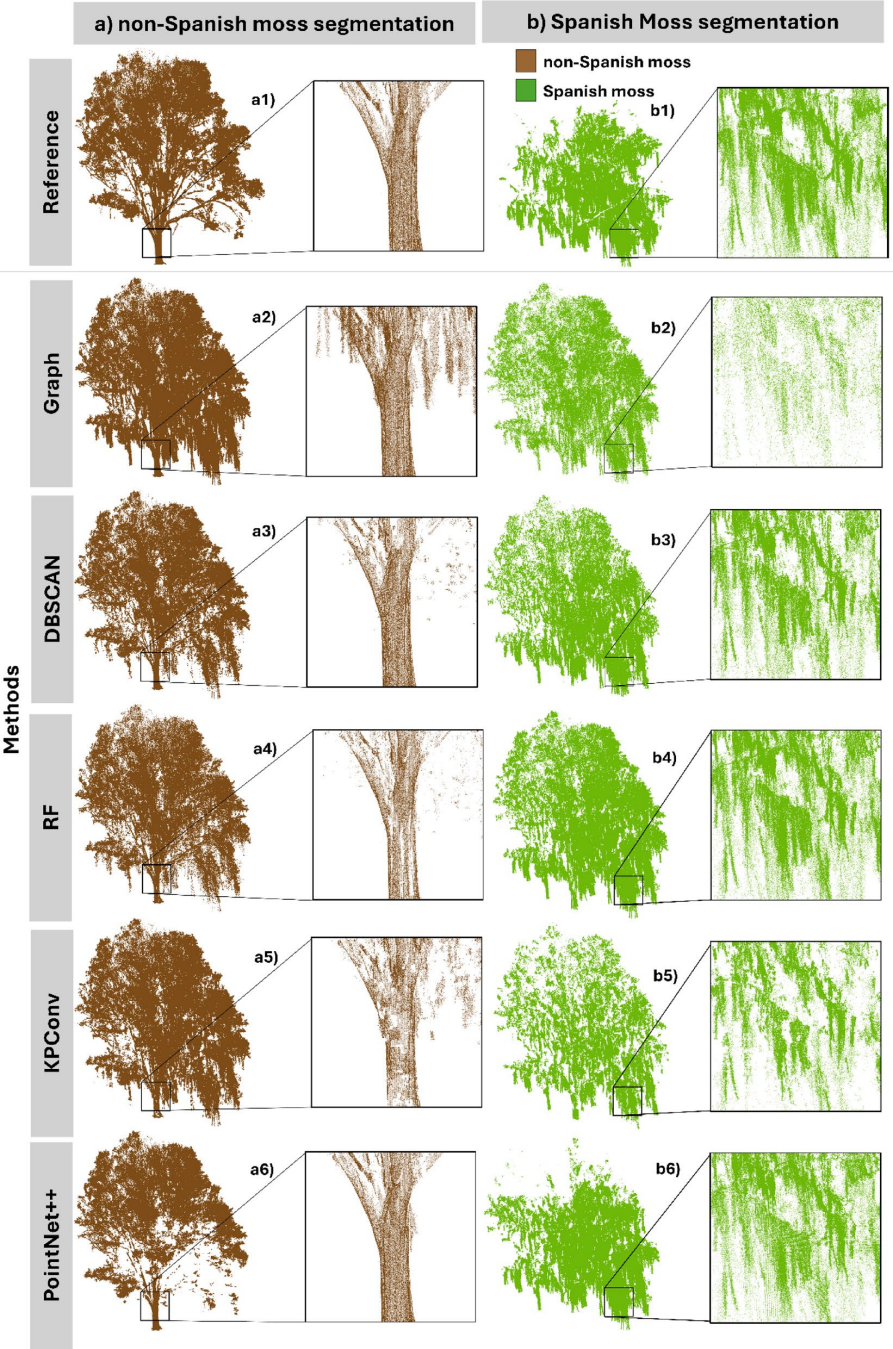
Visual comparison of Spanish moss and non-Spanish moss segmentation for a representative tree across methods, with zoomed-in views relative to the reference.

Visual comparisons on individual trees (Figs. 9, 10) highlight clear differences among methods. Graph consistently overpredicted Spanish moss, failing to recover continuous non-Spanish moss structures. DBSCAN captured more moss patches but frequently mislabeled branches. RF produced dense moss predictions but underrepresented non-Spanish moss areas. KPConv reconstructed coherent woody structures but tended to assign entire branches to moss. PointNet++ most closely resembled the reference, correctly delineating both moss and non-Spanish moss regions, although some branch-to-moss confusion remained.

Per-tree confusion compositions (Fig. 9a) further illustrate these differences. Graph produced high FP, DBSCAN mislabeled many branches as moss, RF achieved high TP but relatively low TN, and KPConv frequently classified whole branches as moss. PointNet++ yielded the highest proportion of TP, with fewer dense FP clusters than other methods. Panels (Fig. 9c1–c4) show typical TP, TN, FP, and FN examples for reference.

### Geometric feature distributions in 3D and importance

**Fig. 11:**
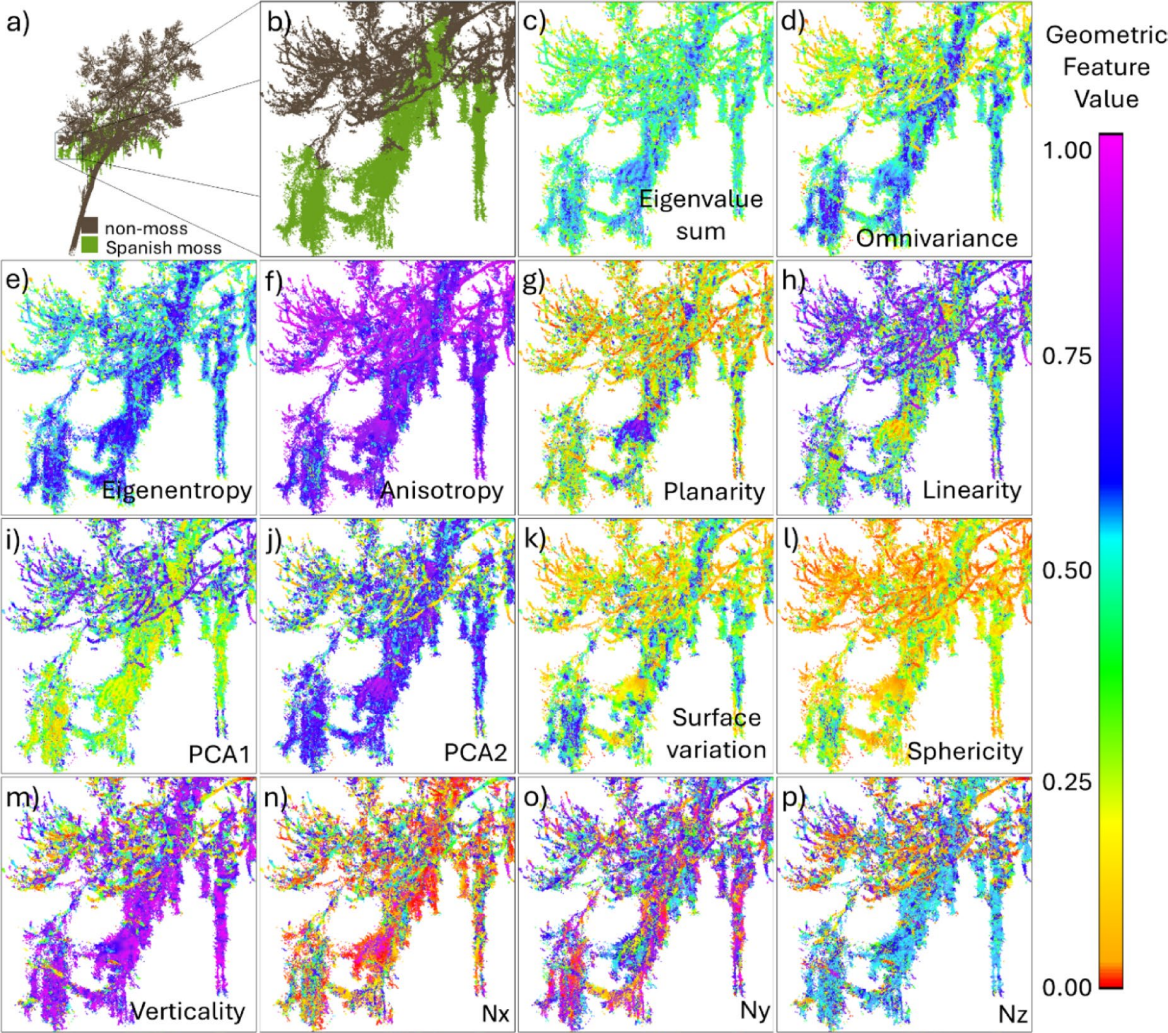
Spatial distribution of 14 geometric metrics on a portion of a tree containing Spanish moss. (**a**) example 3D point cloud with classification; (**b**) zoomed-in view of the classified 3D point cloud; (**c**–**p**) Point-wise distribution of geometric metrics: (**c**) eigenvalue sum, (**d**) omnivariance, (**e**) eigenentropy, (**f**) anisotropy, (**g**) planarity, (**h**) linearity, (**i**) PCA1, (**j**) PCA2, (**k**) surface variation, (**l**) sphericity, (**m**) verticality, (**n**) Nx, (**o**) Ny, and (**p**) Nz.

**Fig. 12:**
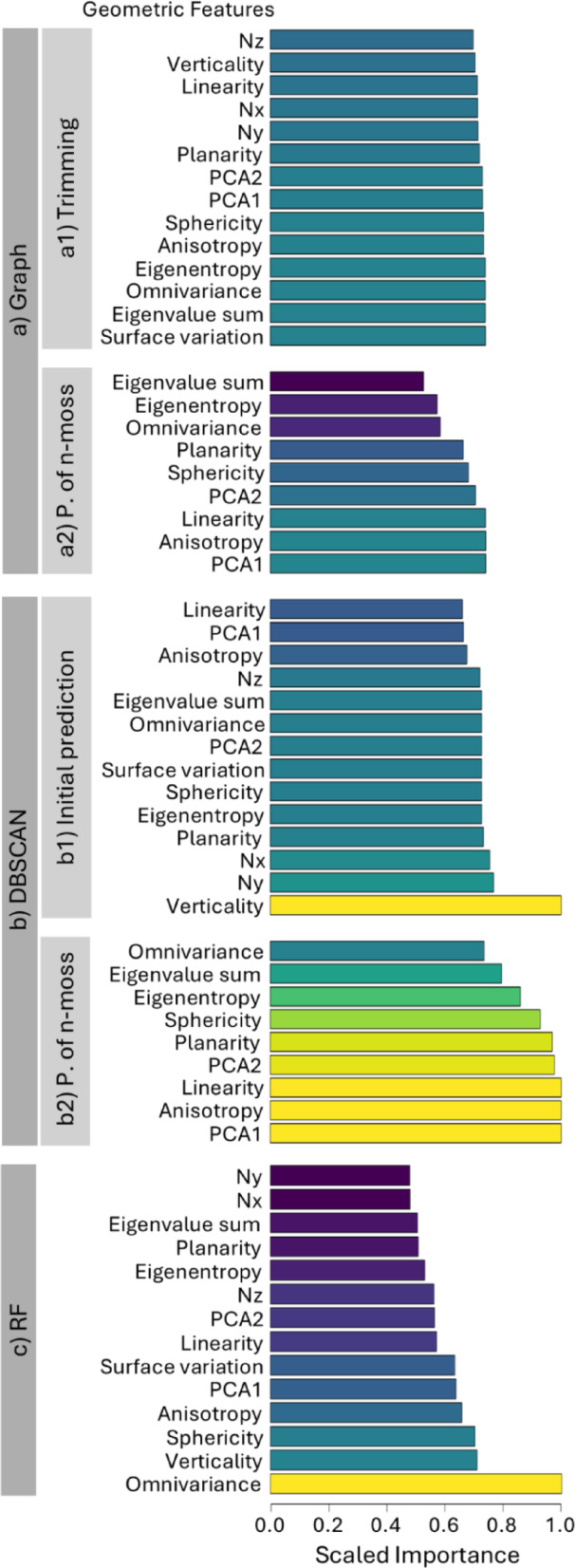
Scaled feature importance of three methods (**a**) Graph, (**b**) DBSCAN, and (**c**) RF. The scaled feature importance of different parts of the Graph method: (a1) trimming and (a2) probability of non-Spanish moss, and the DBSCAN method representing overall accuracy: (b1) initial predictions and (b2) probability of non-Spanish moss. For Graph and DBSCAN scaled feature importance represents overall accuracy and RF scaled feature importance represents mean decrease in gini.

The geometric features used for classification exhibited clear spatial patterns within the point cloud (Fig. 11). Moss and woody structures differed consistently in verticality, sphericity, omnivariance, and the normal vector components (Nx, Ny, Nz). These differences highlight the effectiveness of geometric features as discriminative cues for separating Spanish moss from non-Spanish moss. Feature importance analyses were used to identify the variables that most strongly contributed to the classification performance for each method (Fig. 12). The importance of individual geometric features varied by method and, where applicable, by processing step. In contrast to the other methods, which rely on predefined geometric features, deep learning approaches do not explicitly use handcrafted descriptors. Instead, they learn spatial relationships directly from the point cloud. These relationships are embedded within the network architectures and cannot be expressed as isolated, interpretable features in the same manner as geometric features. Consequently, feature importance analyses do not apply to the deep learning methods.

#### Graph

For the Graph method (Fig. 12a), surface variation was the most influential feature in the trimming step. Eigenvalue sum, omnivariance, and eigentropy also showed significant contributions, while Nz exhibited the lowest importance at this step (Fig. 12a1). When estimating the probability of non-Spanish moss, PCA1 showed the highest importance. Anisotropy and linearity followed in importance, whereas eigenvalue sum contributed the least (Fig. 12a2).

#### DBSCAN

For the DBSCAN method, verticality was the most influential feature during the initial prediction step (Fig. 12b1). The normal vector components Ny and Nx also contributed strongly, while linearity and PCA1 showed minimal influence. In the subsequent probability estimation step (Fig. 12b2), PCA1 exhibited the highest importance, followed by anisotropy and linearity. Omnivariance contributed the least at this stage.

#### Random Forest

**Fig. 13:**
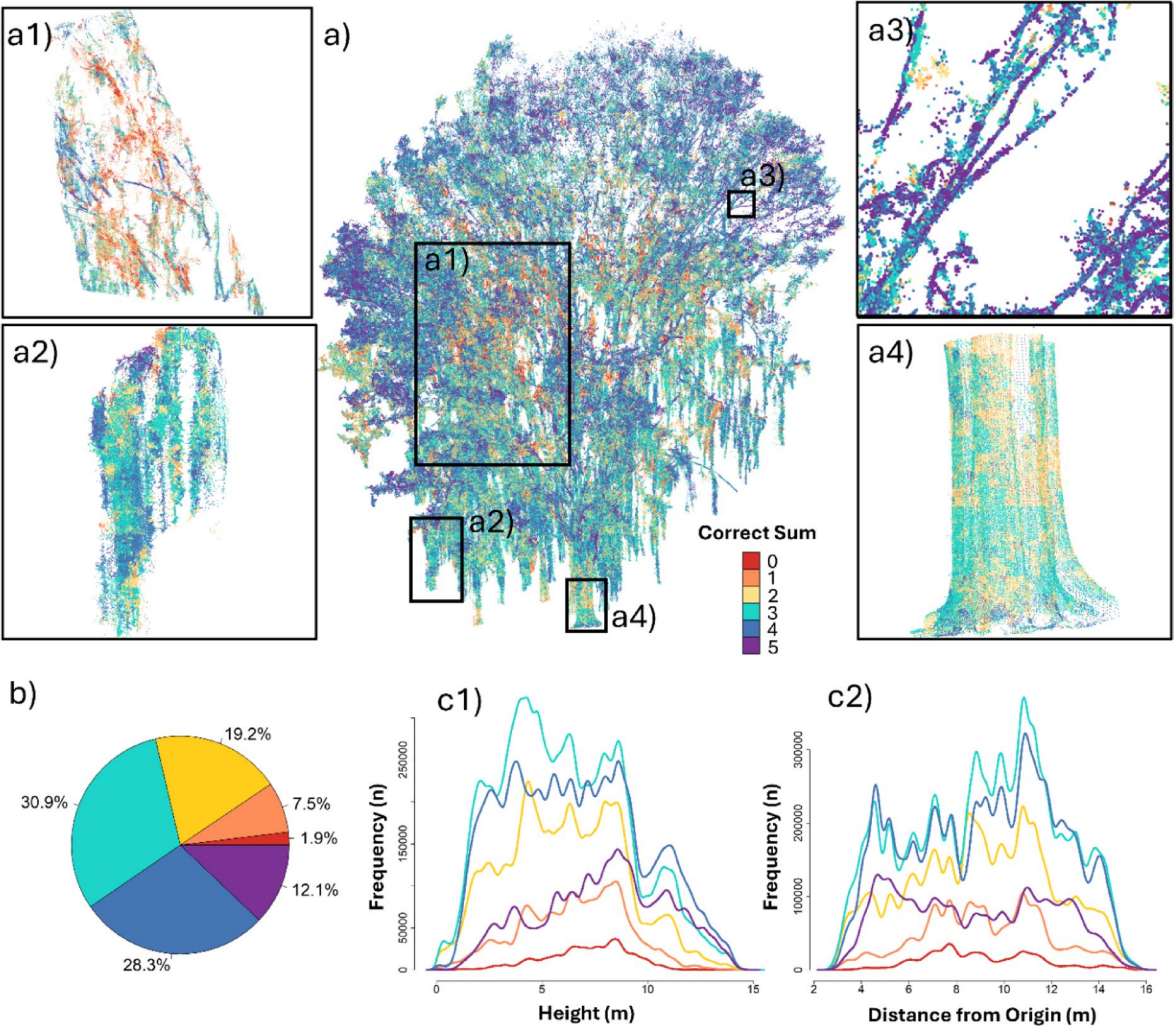
Agreement among methods: (**a**) Number of methods correctly classifying each point; (**b**) Distribution of agreement levels; (c1) Frequency by height; (c2) Frequency by distance from origin.

For the RF method, omnivariance was the most important feature overall (Fig. 12c). Verticality, sphericity, and anisotropy also contributed substantially to model performance. In contrast, Nx and Ny ranked lowest in importance. Collectively, these results demonstrate that local 3D variation and orientation cues are critical for separating Spanish moss from non-Spanish moss. This finding is consistent with the spatial errors observed in Fig. 13.

### Agreement among methods and spatial distribution of errors

The number of methods correctly classifying each point varied across tree structures (Fig. 13a). Branches exhibited the highest agreement, Spanish moss the lowest, and the trunk intermediate. Most points were correctly classified by three to four methods, fewer by all five, and only a small fraction by one or none (Fig. 13b). Frequency distributions by height (Fig. 13c1) and distance from origin (Fig. 13c2) revealed reduced classification accuracy in the central, occluded crown, with improvements toward the outer canopy, though PointNet++ showed some decline at the canopy edge.

### Computational efficiency

Resource requirements varied substantially among methods (Table 5). Training time was longest for KPConv (60 h) and PointNet++ (48 h), moderate for RF (4 h), and zero for unsupervised methods. Prediction time ranged from 2 min (DBSCAN) to 8 h (RF). Memory demands were largest for RF (1024 GB), followed by KPConv (256 GB) and PointNet++ (128 GB). Conversely, unsupervised methods required little memory (Graph: 16 GB; DBSCAN: 8 GB). Thus, PointNet++ offered the highest accuracy but required substantial computational resources, while DBSCAN provided a cost-efficient alternative with moderate performance.

**Table 5:** Computational efficiency of all methods, including training time, prediction time, CPU cores, and memory requirements.

Parameter	Graph	DBSCAN	RF	KPConv	PointNet++
Time to Train (hours)	0	0	3	60	48
Time to Predict (hours)	0.05	0.03	8	4	1
CPU Cores	4	4	16	32	32
memory (GB)	16	8	1024	256	128

## Discussion

This study provides the first application of supervised, unsupervised, and deep learning methods to classify Spanish moss from TLS point clouds. Previous TLS classification research has largely focused on separating leaves and wood^52,53^ or identifying vines and microhabitats^54,55^. By extending classification to an epiphytic species with complex morphology, this work opens a new direction for TLS-based vegetation analysis.

Among the five methods, PointNet++ achieved the highest accuracy (81% on the test set), followed by DBSCAN (70%), KPConv (61%), RF (54%), and Graph (52%). These results confirm the potential of deep learning to outperform traditional methods, while also highlighting systematic errors: KPConv and Graph frequently overestimated moss, RF underrepresented non-Spanish moss, and DBSCAN misclassified branches as moss. Compared with reported accuracies of up to 97% in leaf–wood separation^56^ and ~ 90% completeness for vine detection^54^, the more moderate values found here reflect the added difficulty of Spanish moss classification due to its unordered, filamentous structure.

Agreement among methods was lowest in the central crown and upper canopy, areas prone to high occlusion, echoing similar findings in stem and leaf segmentation studies^55,57^. Feature importance analyses confirmed that orientation and geometric variability were most informative. Surface variation, verticality, and omnivariance were dominant predictors for Graph, DBSCAN, and RF, respectively, while features such as Nz, linearity, and Nx contributed little. These results are consistent with the observation that moss typically hangs vertically, making it challenging to separate from similarly shaped branches. Misclassification may also partly reflect errors in manual labeling, as noted in other TLS classification studies^30^.

While deep learning methods outperformed other methods in accuracy, they required substantially greater computational resources. PointNet++ and KPConv needed 48–60 h of training and large memory allocations (128–256 GB), compared with only 3 h for RF and no training for Graph or DBSCAN. Prediction times were shortest for PointNet++ (1 h) and longest for RF (8 h). These trade-offs are important for field applications where processing large TLS datasets (> 1 million points per scan) is common. In practical terms, PointNet++ offers the most effective balance between accuracy and efficiency, and could be applied as a robust pipeline for Spanish moss detection. DBSCAN also presents a viable alternative in resource-limited settings, offering moderate accuracy at minimal cost.

Despite the strong performance of these methods, several factors should be considered when interpreting the replicability and transferability of the proposed methodology. Achieving comparable classification accuracy benefits from large volumes of TLS data, which impose substantial demands on data storage and processing time. This trade-off between data richness and computational cost is particularly relevant for trees with extensive canopies and complex branching structures, where occlusion can obscure Spanish moss or woody elements and introduce classification uncertainty. In addition, RF and the deep learning methods require considerable computational resources, particularly a large amount of GPU power and RAM, which limit accessibility under resource constraints. The accuracy achieved in this study is also closely tied to the data acquisition strategy, as TLS data were collected during the winter in deciduous trees, minimizing occlusion from foliage. Replication of this approach in a different season or on evergreen trees would require distinguishing Spanish moss from foliage, which can be challenging when leaves occupy similar canopy positions or further increase occlusion. These considerations highlight the importance of clearly defined data collection protocols that account for tree species, canopy architecture, and seasonal conditions. Establishing standardized acquisition guidelines would enhance reproducibility across diverse environments and provide a clearer pathway for extending the proposed methodology to broader forest types and operational contexts.

By demonstrating the feasibility of TLS-based classification of Spanish moss, this study provides the first evidence that epiphyte biomass can be automatically quantified using 3D point clouds. In practical terms, PointNet++ offers a straightforward pipeline, requiring few parameter adjustments and delivering robust classification results, while DBSCAN provides a cost-efficient alternative in resource-limited contexts. Looking ahead, further research should increase the diversity of training data across sites and moss densities to improve generalization and reduce biases from manual labeling. The integration of complementary methods, such as using PointNet++ for high-accuracy moss detection together with DBSCAN for error correction, has the potential to enhance robustness. Importantly, once Spanish moss is reliably separated from woody structures, TLS-derived point clouds can be extended beyond classification to estimate Spanish moss biomass and carbon storage through regression or volumetric modeling. This would provide a non-destructive alternative to traditional destructive sampling^16^ and allow epiphyte dynamics to be incorporated into broader forest monitoring frameworks, contributing to improved assessment of ecosystem function and management practices.

## Conclusion

This study presents the first attempt to use TLS to classify Spanish moss in three-dimensional point clouds, applying unsupervised, machine learning, and deep learning methods. Five methods were evaluated to test their ability to separate Spanish moss from non-Spanish moss structures. The results revealed substantial differences in performance, with PointNet++ providing the highest accuracy, followed by DBSCAN, KPConv, RF, and Graph. These findings demonstrate both the promise and the challenges of extending TLS classification beyond leaves and woody tissues to include epiphytic plants, which present greater complexity due to their filamentous and unordered morphology. Importantly, the ability to reliably separate Spanish moss from woody structures provides a foundation for future studies aimed at quantifying biomass and carbon storage of epiphytes. By establishing a workflow that captures this unique vegetation type, our study highlights the broader potential of TLS and deep learning to advance forest monitoring, ecological research, and management applications.

## Data Availability

The data that support the findings of this study are available from the corresponding author upon reasonable request.
